# Notes and News

**Published:** 1903-07-04

**Authors:** 


### NOTES AND NEWS.

The Princess Louise will visit the Hospital and Home for Jewish Incurables on Friday, and on the same day the new library at Guy's Hospital will be opened by Sir F. Will, M.P.

During his visit to England President Loubet will visit the French Hospital.

On Thursday, July 9th, a costume ball will be given under Royal Patronage, in aid of Charing Cross Hospital, at the Royal Albert Hall.

Amongst the medical recipients of Birthday Honours are the following: Mr. A. Fripp, C.V.O., C.B.; Stephen Mackenzie, Esq., M.D.; E. C. Perry, Esq., M.D.; and P. Heron Watson, Esq., M.D., LL.D., F.R.S., who have been created knights. Surgeon-General G. Evatt has received the Order of the Bath, Military Division. Patrick Manson, Esq., M.D., C.M.G., has been created a Knight of the Order of St. Michael and St. George.

For some time past the Royal Hospital for Women and Children near Waterloo Station has adopted a novel form of appeal, in the shape of a captive balloon, setting forth in large letters on it the need of £5,000 to rebuild the hospital. Apparently the balloon felt the catholic claims of the institution, so leaving its confined quarters, it sailed away to new fields where funds might be obtainable. It was eventually found in Essex.

The committee entrusted with the rebuilding scheme of St. Bartholomew's Hospital by the Lord Mayor's committee of inquiry held a meeting last week, and decided that as the new out-patients' department was the most necessary part of the building scheme, it should be proceeded with first. The cost is estimated at £100,000. Mr. I'Anson, the architect, submitted alternative schemes for the whole rebuilding, and these with the medical council's reports were considered by the committee. A report from the committee may be expected after the next meeting of the Lord Mayor's committee and the building committee together.

The last report of the Medical Officer of Health for the City is encouraging in respect to the food of the Metropolis. Out of 82 samples of food and drugs submitted to the analyst, only one was adulterated. The inspection of meat, both in slaughter-houses and markets, continues to be very necessary, as shown by the amount seized as unfit for consumption.

We do not envy the sanitary inspectors in some of the Metropolitan districts which have suffered from the recent floods. Some of the poor habitations are in a terrible condition, and one which is not likely to be improved by the sudden heat, which, whilst it dries damp quarters, aggravates the evil of foul smelling places, and may produce a good many ailments of varying severity.

The next competition of the "Howard Medal" of the Royal Statistical Society will be held in 1904. The subject is "The Effect, as shown by Statistics, of British Statutory Regulations directed to the Improvement of the Hygienic Conditions of Industrial Occupations." The successful competitor will receive £20 as well as the medal. The essay must not exceed 100 pages of about 500 words. The competition is open to all, and particulars may be obtained of the Secretary, 9 Adelphi Terrace.

A very superficial knowledge of the laws of life and health is necessary to convince dwellers in the Metropolis that open spaces are of vital sanitary importance to the population. The fact is so generally admitted that any scheme to form new lungs for London or retain the existing air spaces meets with hearty support. The projects are expensive, but they are judged worth the cost. The latest proposal is one to extend and help to preserve Hampstead Heath at a cost of £48,000. New railways are forming which will bring more and more Londoners away from their crowded homes, to the benefit of both the inhabitants and the habitations, which are left for a few hours tenantless. But crowds mean destruction to vegetation, and so it is necessary to have more space on which holiday seekers can distribute themselves. We hope that Londoners will co-operate to secure a benefit which directly or indirectly affects the majority of them.

The municipal lodging-house is a very useful institution, and the superior advantages which it offers in the way of comfort and cleanliness are really helping to make it popular among those who patronise it. It would be a pity to jeopardise its popularity by any too autocratic course of procedure. It must win its way by its own merits, as other hotels of greater pretensions have to do, and, in ordinary circumstances, avoid the chance of alienating its patrons. In times of epidemic the sanitary authorities may permissibly take stronger measures, but even then a bribe may answer better than too obvious a show of authority. During a small-pox scare a few years ago the sanitary authorities in Glasgow gave a week's free board and lodging to any of the residents in the municipal or other common lodging-houses who would submit to be vaccinated. They were thus kept under observation till it was seen if the operation was successful, and felt that they were making a profit out of it.

